# Duplex Fluorinated and Atomic Layer Deposition-Derived ZrO_2_ Coatings Improve the Corrosion Resistance and Mechanical Properties of Mg-2Zn-0.46Y-0.5Nd (wt.%) Alloy Plates and Screws

**DOI:** 10.3390/ma17143485

**Published:** 2024-07-14

**Authors:** Tiancheng Qiu, Rong Yang, Liangwei Chen, Guanqi Liu, Jianmin Han, Chuanbin Guo

**Affiliations:** 1 Department of Oral and Maxillofacial Surgery, Peking University School and Hospital of Stomatology, Beijing 100081, China; 18612882973@163.com (T.Q.); chenliangwei@bjmu.edu.cn (L.C.); 2 Department of General Dentistry, Peking University School and Hospital of Stomatology, Beijing 100081, China; yrpkuhsc2011@163.com; 3 National Engineering Laboratory for Digital and Material Technology of Stomatology, Department of Dental Materials, Peking University School and Hospital of Stomatology, Beijing 100081, China; 18811321319@163.com

**Keywords:** internal fixation, Mg-Zn-Y-Nd alloy, degradation, fluorinated coating, atomic layer deposition, stress

## Abstract

This study investigated the corrosion resistance and mechanical properties of Mg-2Zn-0.46Y-0.5Nd (wt.%) alloy plates and screws with fluorinated coatings and atomic layer deposition (ALD)-derived zirconia (ZrO_2_) coatings in vitro under physiological stress conditions. Synthetic polyurethane hemimandible replicas were split and fixed as the following three groups of magnesium alloy plates and screws: no additional surface coating treatment (Group A), with fluorinated coatings (Group B), and with duplex fluorinated and ALD-derived 100 nm ZrO_2_ coatings (Group C). A circulating stress of 1–10 N was applied to the distal bone segment, and a 4-week simulated body fluid immersion test was employed to study the remaining material volume and the mechanical properties of the different groups. Compared with Group A and Group B, the degradation rate of magnesium alloy plates and screws’ head regions was significantly slowed down under the protection of duplex MgF_2_/ZrO_2_ coatings (*p* < 0.01). There was no significant difference in the degradation rate of the screw shaft region between groups (*p* = 0.077). In contrast to fluoride coatings, duplex MgF_2_/ZrO_2_ coatings maintained the mechanical strength of magnesium alloy plates and screws after a 14 day in vitro SBF immersion test. We conclude that duplex MgF_2_/ZrO_2_ coatings exhibited a certain protective effect on the Mg alloy plates and screws under physiological stress conditions.

## 1. Introduction

Ridge internal fixation is the main surgical procedure for treating maxillofacial fractures [1]. In the past few decades, titanium alloys have been used as the main material for manufacturing rigid internal fixation plates and screws owing to their good biocompatibility and the ability to provide sufficient mechanical strength for fracture healing [2,3]. However, due to stress shielding, interference in radiological imaging examination, and local rejection actions, many patients require a second surgery for the removal of the titanium plates and screws [4,5]. Biodegradable polymer-based osteosynthesis materials can be used as an alternative to rigid internal fixation, which degrades spontaneously after completing its function [6,7]. However, the mechanical strength of polymer devices is not adequate for load-bearing applications [8,9]. Furthermore, acidic degradation products are continuously produced during the degradation of polymer materials, which causes foreign body reactions and affects tissue healing [10,11]. Therefore, finding a degradable material that is biocompatible and can provide sufficient strength is clinically important.

Magnesium has an elastic modulus similar to that of the cortical bone. It avoids stress-shielding effects and provides sufficient strength compared with polymer-based materials [12]. Moreover, Mg ions can promote subperiosteal osteogenesis by stimulating the related cytokine regulatory pathways in the periosteum, thereby accelerating fracture healing [13,14,15]. Osteosynthesis implants using Mg screws and pins have already been used in clinical surgery [16]; however, the application of Mg alloy plates and screws is still stagnant in animal experiments [17,18,19]. Compared with intraosseous implants, the in vivo environment of plates and screws is more complicated, and it is mostly used for fracture fixation in regions with greater biological stress [17,20]. Previous studies have shown that after implantation of Mg alloy plates and screws for the rigid fixation of LeFort I osteotomy in beagle dogs, the plates were absorbed rapidly and separated from the bone surface, which affected the strength of internal fixation and led to local inflammation and foreign body reactions [21].

To address the difficulties during the application and achieve the regulated degradation rate of Mg alloy, magnesium alloys of different elements compositions and different surface modification methods have been applied in vitro and in vivo [22,23].

Mg alloying methods can alter the microstructure and reduce the particle size of the Mg-related material, thereby improving the biomechanical and corrosion properties [24,25]. Rare-earth-, zinc-, aluminum-, and calcium-based Mg alloys have been widely analyzed in previous in vitro and in vivo studies [21,26,27,28,29]. The addition of zinc and rare earth elements to magnesium alloys can enhance the mechanical properties and corrosion resistance of magnesium alloys [23]. The Mg-2Zn-0.46Y-0.5Nd (wt.%) alloy developed by our research team was applied as a guided bone regeneration membrane implanted in beagle dogs, which showed good corrosion resistance and mechanical strength [30]. Furthermore, the content of rare earth elements of this Mg alloy is only one-third of that of WE43 (a commonly used Mg alloy), which indicates a better biocompatibility [30].

Surface coating methods can be characterized as conversion coatings, deposited coatings, or a combination of the two [22]. Fluorinated coating is a widely applied conversion coating that forms MgF_2_, instead of the original oxide layer, on the surface of the Mg alloys, which can slow down the degradation of Mg alloys [31]. Nevertheless, microcracks appear in fluorinated coatings during the degradation process, causing more pitting corrosion compared with those that are not protected by coatings. Deposited coatings refer to the coatings whose substrates are not involved in coating formation. Atomic-layer deposition (ALD) is a deposition technique used to deposit nanoscale thin films on substrates [32]. Even though ALD provides a uniform, pinhole-free, and thickness-controlled coating, ALD-derived coating is generally regarded as the outmost layer for the weaker bond strength with the basal Mg alloy [22]. A combined application of conversion and deposited coatings may offer the advantage of both coatings and minimize the rate of degradation [31]. Presently, fluoride and plasma electrolytic oxidation coatings were modified on the Mg plate–screw system in vivo to evaluate the protective effect of the coatings on the material after implantation [18,19]. However, no bone fracture model was designed, and Mg alloys plates and screws were not subjected to physiological stress. To the best of our knowledge, no study has evaluated the protective effect of coatings on substrate Mg plate–screw systems under physiological stress.

Therefore, the present study was designed to evaluate the protective effect of combined fluorinated and ALD-derived zirconia (ZrO_2_) coatings on the substrate Mg alloy plate–screw system under physiological force stimulation.

## 2. Materials and Methods

### 2.1. Specimen Preparation

The Mg-2Zn-0.46Y-0.5Nd (wt.%) applied in this study was provided by the Materials Research Center, Zhengzhou University. Plates and screws were manufactured by Cibei Medical Treatment Appliance Company (Ningbo, China). The length, width, and thickness of the plates were 20.00 mm, 4.00 mm, and 1.00 mm, respectively; the diameter and length of the screws were 2 mm and 6 mm, respectively, which conforms to the standard size of implants in maxillofacial surgery. All plates and screws were cleaned ultrasonically using ethanol and acetone and sterilized with ultraviolet irradiation (UV lamp, predominantly 254 nm) for 30 min before they were used in vitro.

**Figure 1:**
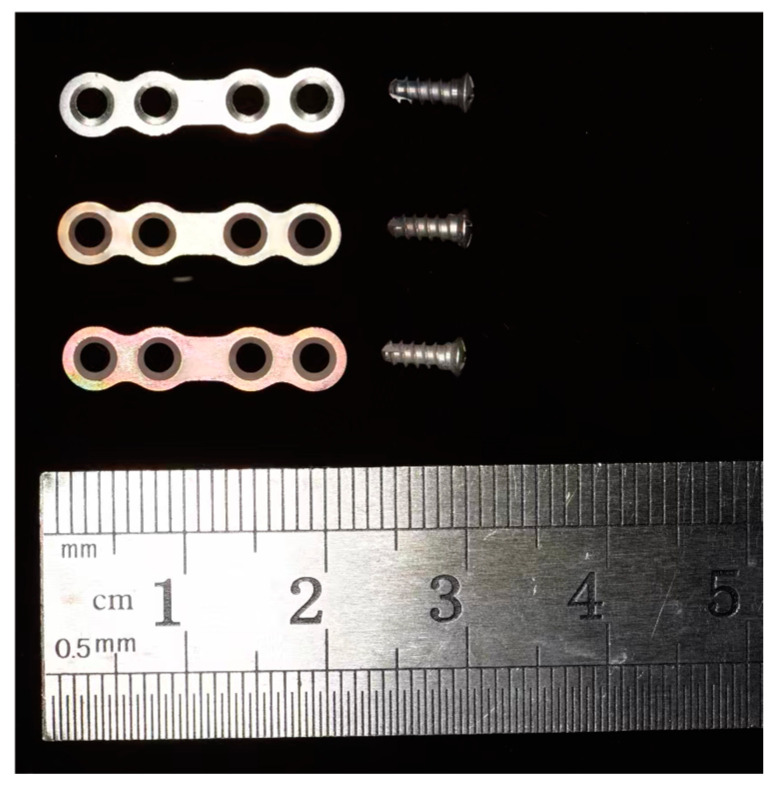
Three types of magnesium alloy plates and screws. Up: Group A, without surface coatings. Middle: Group B, with the fluoride coating. Bottom: Group C, with the duplex fluorinated and ZrO_2_ coatings.

The Mg alloy plates and screws were divided into three groups (Figure 1). Group A comprised Mg alloy plates and screws without additional surface coatings. Plates and screws in Group B were subjected to fluoride treatment. They were first treated in boiled NaOH solution (1 mol/L) for 4 h and then allowed to react with an HF acid solution (20 wt.%) at room temperature for 2–6 h to form a uniform magnesium fluoride (MgF_2_) layer on the surface of the substrate Mg alloy. Plates and screws in Group C had duplex MgF_2_/ZrO_2_ coatings. First, a MgF_2_ coating was obtained on the sample, and then a ZrO_2_ coating was deposited using an ALD reactor (ALD, Ensure Nanotech, Labnano 9100, Beijing, China). Zirconium tetrakis (dimethylamido) (TDMAZ) was used as the precursor, and O_3_ was used as the oxidant. N_2_ was applied to remove residual reactants and byproducts from the chamber. The deposition temperature was set at 150 °C, and each cycle was performed in the following two parts: a 200 ms TDMAZ precursor pulse, followed by a 60 s high-purity N_2_ purge (flow rate of 20 scc), and a 150 ms O_3_ precursor pulse, followed by a 10 s high-purity N_2_ purge. After 900 cycles, a 100 nm zirconia (ZrO_2_) coating was obtained on the sample surface.

### 2.2. Microstructure and Surface Characterization

A field emission scanning electron microscope (SEM, HITACHI SU8220, Tokyo, Japan) with a Bruker FlatQuad X-ray spectrometer (Bruker, Billerica, MA, USA) was used to investigate the microstructure—the distribution of elements on the surface of plates and screws in groups A–C. Cross-sections of Group C samples were observed to investigate the extent and uniformity of the duplex MgF_2_/ZrO_2_ coatings. The accelerating voltage and working distance were set to 15.0 kV and 15.0 mm, respectively. The phase of all sample surfaces was determined using X-ray diffraction (XRD) spectra. The scanning was performed using Cu K*α* radiation as the source, with 2*θ* in the range of 10°–90° and a rate of 2°/min, along with a 1° glancing angle. Phase analysis was performed using the standardICDD (PDF-4+, 2019).

### 2.3. In Vitro Experiment

**Figure 2:**
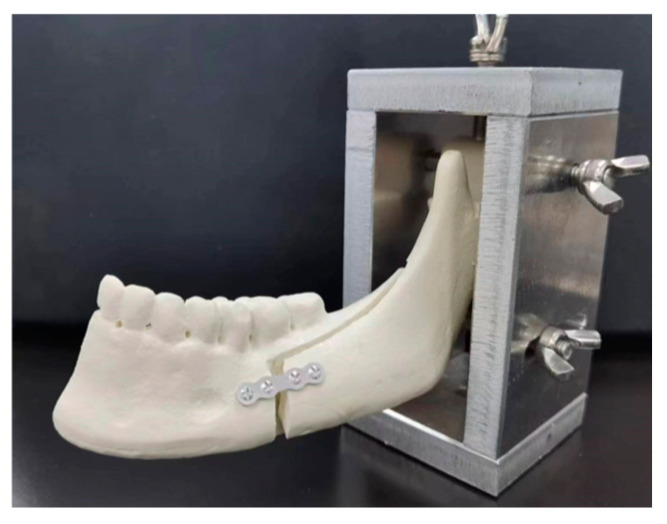
Hemimandible rigidly fixed by Mg alloy plates and screws were stabilized in the support apparatus.

Synthetic polyurethane hemimandible replicas were used in this study to simulate the structure of the mandible, as described in a previous study [33,34]. After sagittal split ramus osteotomy (SSRO), the distal segment was rigidly fixed at a 5 mm setback position. The plate–screw system was carefully mounted on polyurethane hemimandible replicas without destroying the surface coating. For this purpose, the screw holes were first drilled and subsequently tapped to reduce the torque required for screw fixing. All screws were inserted perpendicular to the replica surface, whereas the plates were positioned parallel to the occlusal plane. Hemimandibles of all groups were stabilized in the support apparatus. The condyle and ramus of the proximal segment were fixed to avoid movement, whereas the distal segment could move freely (Figure 2).

**Figure 3:**
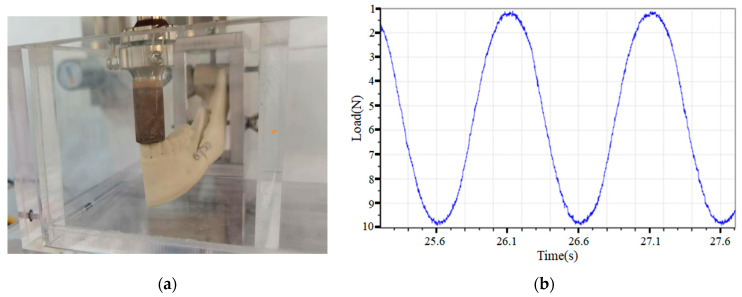
Loading forces were applied on the incisor edge (**a**). The frequency of the stress cycle was 1 Hz, and the loading force was varied from 1–10 N (**b**).

The universal testing machine Electropuls 3000 (Instron, Norwood, MA, USA) was used for fretting corrosion tests. All the hemimandibles were immersed in a simulated body fluid (SBF), and the pH was controlled at 7.35–7.50. To simulate the bite forces recovered from orthognathic surgeries, a varied 1–10 N vertical compression force was applied to the incisor edge (Figure 3a). The stress force was used according to a previous in vitro study, which can be withstood by the titanium alloy internal fixation system [33]. The loading frequency was 60 cycles/min, which was maintained for 30 h and 108,000 cycles (Figure 3b). The hemimandibles fixed by different fixation groups were continuously immersed in SBF at 37 °C for 4 weeks. The solution was changed every four days, and pH was controlled at 7.35–7.50 every day.

### 2.4. Micro-Computed Tomography Analysis

The samples of all groups were scanned using micro-computed tomography (micro-CT) (Siemens, Munich, Germany) before and after their immersion in the SBF solution. The images were acquired at an effective pixel size of 8.82 μm, an accelerating voltage and filament current of 80 kV and 500 μA, respectively, and an exposure time of 1500 ms in each of the 360° rotational steps. Inveon Research Workplace software 4.2 (Siemens, Knoxville, TN, USA) was used to analyze the degradation of the plate–screw system.

### 2.5. Loading Test

A loading test was performed at the following five time points: T0, before the experiment; T1, after 108,000 cycles of alternating forces; T2: after 3 days of immersion; T3, after 7 days of immersion; and T4, after 14 days of immersion. Using the universal testing machine Electropuls3000 (Instron, Norwood, MA, USA), the loading forces were transmitted to the incisal edge, which displaced the distal segment linear at a rate of 1 mm/s. The loading force for the incisal displacement of the distal segment at 1 mm, 3 mm, 5 mm, and 10 mm were measured, and the means with standard deviations of the data were calculated.

### 2.6. Statistical Analysis

SPSS 23.0 (SPSS Inc., Chicago, IL, USA) was used for data analysis. Levene’s test was performed to evaluate the homogeneity of variances. Analysis of variance (ANOVA) was used to evaluate the loading force of different Mg alloy fixation groups at the different incisal displacements and different time points and the residual volume of plates and screws of different fixation groups after in vitro experiments. A *p* value of less than 0.05 was considered to denote a statistically significant difference.

## 3. Results

### 3.1. Microstructure of the Samples

**Figure 4:**
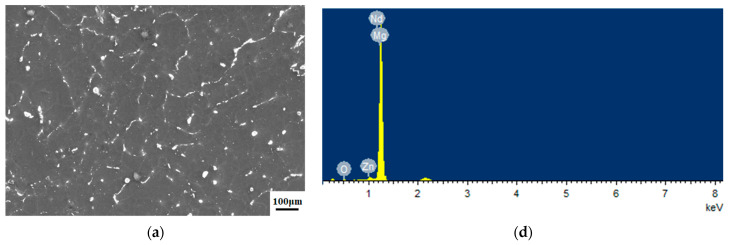

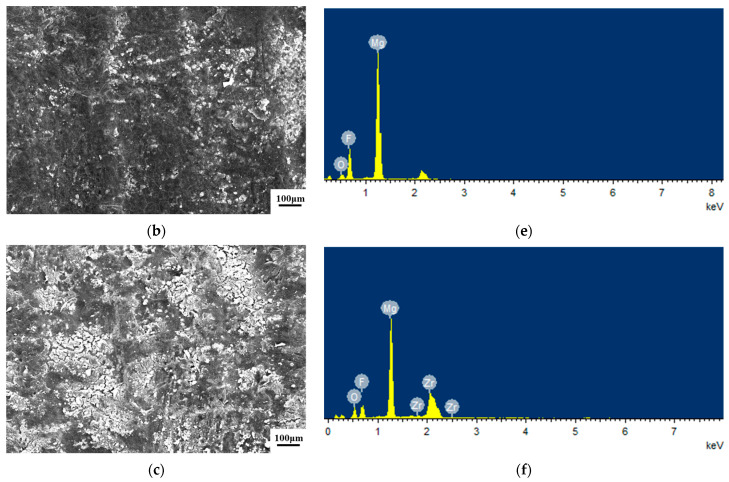
Scanning electron microscopy images and energy-dispersive X-ray images of Mg-Zn-Y-Nd alloy (**a**,**d**), fluorinated coatings (**b**,**e**), duplex MgF_2_/ZrO_2_ coatings (**c**,**f**).

**Figure 5:**
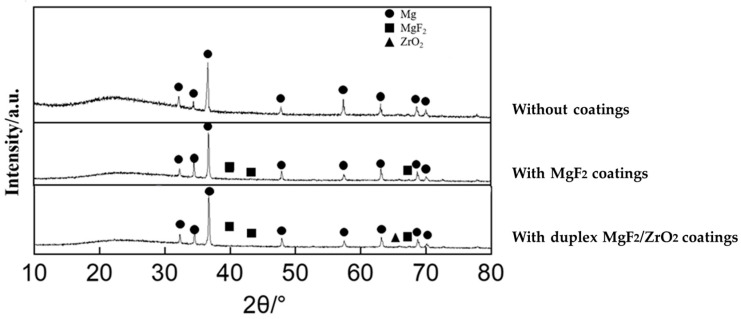
X-ray diffraction patterns of Mg alloy without coatings, Mg alloy with MgF_2_ coatings, and Mg alloy with duplex MgF_2_/ZrO_2_ coatings.

**Figure 6:**
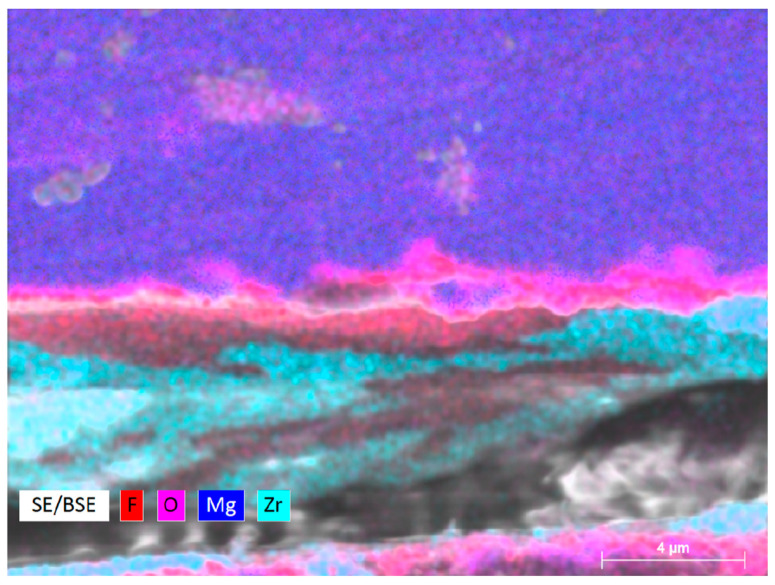
Field emission scanning electron microscopy images showing cross-section of duplex MgF_2_/ZrO_2_ coatings.

Surface morphologies of the samples in the different groups are shown in Figure 4a–c. The results of energy-dispersive spectroscopy (EDS) showed that the fluorinated and ALD ZrO_2_ coatings were successfully applied to the Mg alloy surface (Figure 4d–f). The XRD pattern of the test samples are shown in Figure 5. According to standard JCPDS data, diffraction peaks for the formation of MgF_2_ were found at 2θ = 40.4°, 43.7°, and 67.7°, whereas that for the formation of ZrO_2_ crystalline anatase was observed at 2θ = 65.8°. Cross-sectional morphology of Group C clearly presented with the interfaces of outer ZrO_2_, intermediate MgF_2_, and Mg substrate (Figure 6).

### 3.2. Degradation of the Plate–Screw System

**Figure 7:**
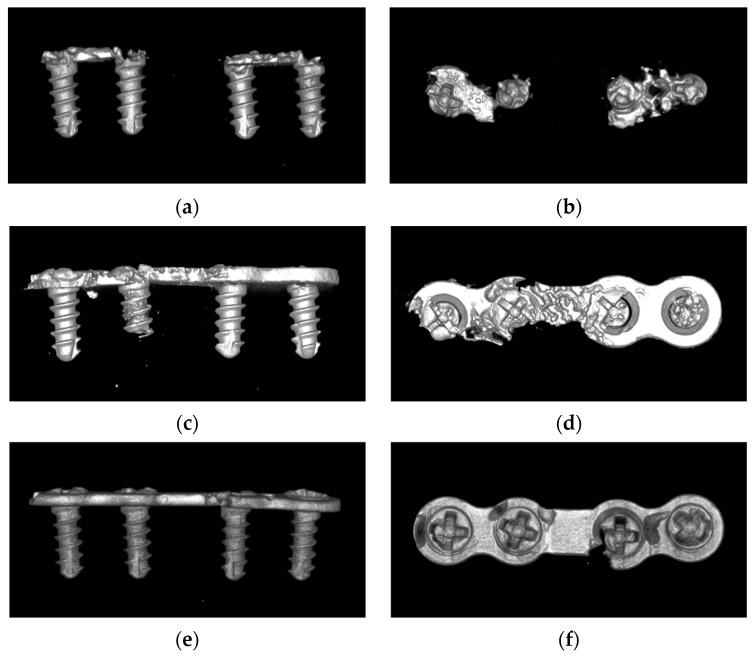
Micro-CT scan of degradation of Mg alloy plates and screws in the different groups after the 4 week in vitro experiment. ((**a**,**b**): Without surface coatings. (**c**,**d**): With the fluorinated coating. (**e**,**f**): With the duplex fluorinated and ALD ZrO_2_ coatings.).

The degradation of all groups of the plate–screw system was studied after the 4 week in vitro experiment by using micro-CT (Figure 7). The residual volume of the plates and screws of the different groups are presented in Table 1.

**Figure 8:**
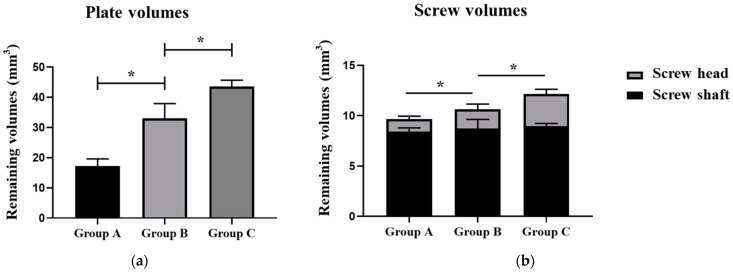
Micro-CT analysis of the remaining volumes of the Mg alloy plates and screws in the different groups after the 4-week in vitro test. (**a**) Comparison of the remaining plate volume; (**b**) Comparison of the remaining screw volume. Group A, without surface coatings; Group B, with the fluorinated coating; Group C, with the duplex fluorinated and ALD ZrO_2_ coatings. *, *p* < 0.05.

The degradation rate of alloy plates varied significantly among the different groups. The results showed that the remaining volume of magnesium alloy plates in Group C was significantly larger than that in Group B. Group A has no protective coating on the surface, and the remaining volume of the magnesium plate was significantly smaller than that of Group B and Group C (Figure 8a).

Corrosion behaviors varied between the different regions of the screws (Figure 8b). The degradation rate of the screw shaft was relatively low, with no significant difference among the different groups. Conversely, the degradation rate of the screw head varied significantly between the different groups. In Group A, 68.51% volume of the screw head was degraded. Furthermore, the fluorinated coatings could not sufficiently protect the screw head from degradation, and its degradation volume was more than 50% within 4 weeks. The duplex MgF_2_/ZrO_2_ coatings significantly slowed down the degradation of the screw head, maintaining the screw head degradation volume at more than 80%.

### 3.3. Loading Test

**Figure 9:**
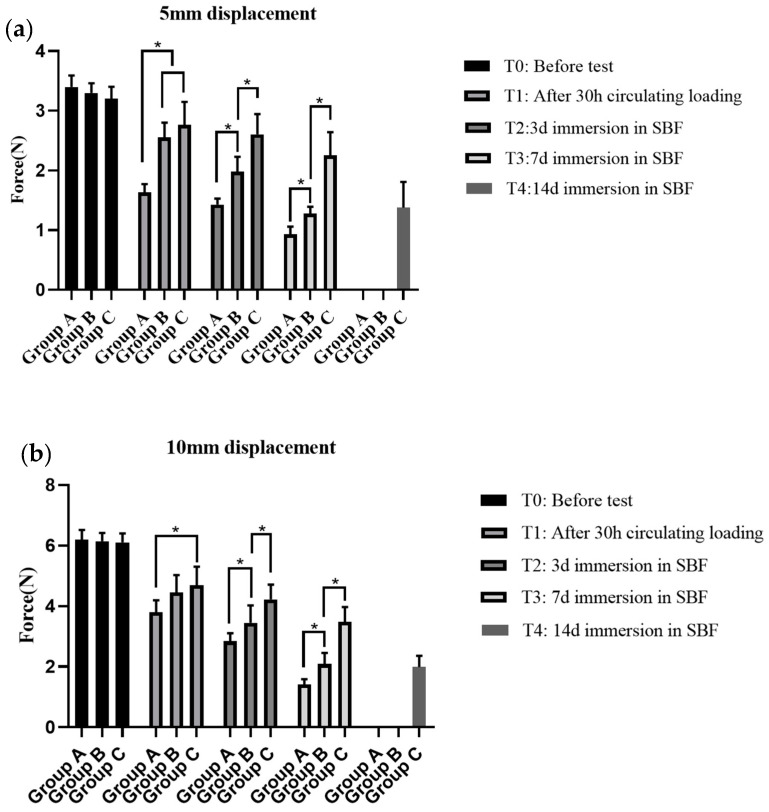
Comparing the loading forces of the different groups of the magnesium alloy plate–screw systems at different time points. ((**a**). 5 mm displacement; (**b**). 10 mm displacement). Group A, without surface coatings; Group B, with the fluorinated coatings; Group C, with the duplex fluorinated and ALD ZrO_2_ coatings. *, *p* < 0.05.

Loading forces that the different groups of the plate–screw system could withstand at the displacement of 1 mm, 3 mm, 5 mm, and 10 mm at T1–T4 are presented in Table 2. Figure 9 shows the comparison of loading forces that the different groups could afford at a displacement of 5 mm and 10 mm. The compressive stress that Group A could withstand decreased rapidly at the beginning of the test, remaining with only about 50% of the original mechanical strength at the T2 time point and broking down between the T3–T4 time points. Group C showed more stable mechanical properties than Group B, and its mechanical strength remained more than 50% at the T3 time point. Group B maintained the overall strength of the material in the early stage; however, with the increasing degradation time, the compressive stress that it could withstand decreased significantly. The plate–screw system in groups A and B was spontaneously broken from the bridge region of plate between the T3 to T4 time points, while the plate–screw system in Group C lost mechanical properties during the 14–21 days degradation period.

## 4. Discussion

Controlling the early degradation rate, delaying local hydrogen production, and preventing serious damage and local infection in the early stage of implantation are necessary for successfully applying Mg alloy plate–screw systems in vivo [17,18,21]. Previous studies have shown that the accelerated degradation rate of Mg alloys due to compressive stress renders their application to fracture fixation in more stressed regions challenging [20,21,35]. Therefore, we, for the first time, analyzed the degradation rate of Mg alloy plate–screw systems under physiological stress.

The rapid corrosion of Mg alloys in the biological environment has been the most prominent problem that needs to be addressed. Surface coatings can not only enhance the corrosion resistance but also provide additional biological functions [36]. Fluoride coatings are the commonly used chemical conversion coatings with good compactness, low water solubility, and high adhesive strength [31,37]. Moreover, fluorine exists in body fluids and can regulate Ca and P metabolism and deposition, which are conducive to osteogenesis [38]. Nonetheless, the protective effect of fluoride coatings on Mg alloys is controversial. Naujokat et al. found that the fluoridated surface had no effect on the in vivo degradation of the plate–screw system [19]. Witte et al. demonstrated that MgF_2_ coatings mitigated the in vivo corrosion rate of Mg alloys; however, pitting corrosion was found on the substrate surface [39].

Deposited coatings are advantageous because they can exhibit enhanced corrosion resistance and function [36]. However, owing to their poor binding strength to substrates, deposited coatings are generally used for coating the outermost layers [40]. ALD, one of the deposited coating techniques, has gradually attracted researchers’ attention for its good reproducibility, pinhole-free nature, conformality, and accurate thickness control [41]. Different oxidative ALD coatings have been evaluated for their corrosion and cytotoxicity performances [42]. TiO_2_ and ZrO_2_ are important biomaterials that are widely used in dental implants and ceramic crowns [43]. Ti is biocompatible and can stimulate in vivo osteoconductivity; however, the poor corrosion resistance of TiO_2_ coatings limits its applications as a protective coating [44]. Daubert et al. demonstrated that ZrO_2_ and HfO_2_ exhibited better corrosion resistance and cell viability than TiO_2_, thus indicating a strong correlation between biocompatibility and corrosion resistance [42]. Therefore, we selected the 100 nm ZrO_2_ ALD coating as the outer protective coating to analyze its protection performance under stress conditions.

After a 4-week in vitro experiment, different regions of the whole rigid internal fixation system exhibited different degradation characteristics. The screw shaft region exhibited a significantly lower corrosion rate than the plate and screw head region. Moreover, the degradation rate of the screw shaft region was not affected, irrespective of the presence or absence of surface coatings. This phenomenon has been reported in a previous in vivo study and can be attributed to the simple degradation environment around the screw shaft [17]. This region was protected by synthetic polyurethane replicas in vitro and by the bone marrow in vivo from contact with body fluids, which provided a stable degradation environment, thereby ensuring its early structural integrity [17,35]. Therefore, the surface coating of the screw shaft can be selected to promote osseointegration and osteogenesis, irrespective of its weak protective effect that may lead to early rapid damage.

Conversely, we found that compared with the screw shaft region, the plate and screw head were rapidly corroded. The reasons can be as follows: (a) The plate and screw head were in contact with the flow simulation body fluid, and a stable corrosion layer could not be formed on the surface [17]; (b) Due to the compressive stress between the screw head and the plate when installing the screw and the stress between the plate and the screw head when simulating the chewing movement, the surface structure of the Mg alloy material underwent constant destruction and the degradation rate was accelerated. Similarly, Wolters et al. conducted an in vitro test using the LAE442 Mg alloy to fabricate the plate and screw and found that the corrosion rate increases with an increase in the compressive stress [35]. (c) Crevices existed in the bone–bone plate interface and in the contact area between the bone plate and the screw. The accumulation of degradation products in these crevices accelerated the degradation of the proximity material, making the surface of the material porous and accelerating the degradation rate. Wu et al. verified this phenomenon using an ingenious in vitro experimental design [45].

Different surface coatings show different protective effects on the substrate Mg alloy. Compared with Group A, the fluorinated coating in Group B had a significant protective effect on the magnesium alloy plate and screw head, and the corrosion rate was delayed to some extent. However, the lack of uniformity of the fluorinated coating limited its protective effect, and during the degradation process, the degradation of Mg plates and screws in Group B was found to be nonuniform and from local rapid degradation failure. Compared with fluorinated coating alone in Group B, the compactness and homogeneity of the ALD ZrO_2_ coating in Group C provided a stronger protective effect and delayed the degradation time of the Mg alloy. After the 4-week in vitro immersion test, the morphological structure of the screw head and plate in Group C was relatively completely retained.

Compared with micro-CT analysis, the results of the loading tests can directly reflect the remaining mechanical properties of the Mg alloy plates and screws. To realistically mimic the loading force of the plate and screw system fixed on the mandible, we used an SSRO model, as described by Oh et al. in an in vitro experiment [33]. Based on the stress that the titanium plates and screws could withstand and to mimic the occlusal force within 1 month of orthognathic surgery, a 1–10 N dynamic stress was applied on the incisal edge. In this study, the magnitude of stress that the Mg plates and screws could withstand at different time points during the in vitro experiment reflects the change of their mechanical properties.

Group A Mg plates and screws showed rapid degradation during cyclic loading and after immersion in SBF. During cyclic loading degradation, corrosive layers formed on the surface of the Mg alloy were not stable and were continuously destroyed by the cyclic loading force, thereby failing to provide sufficient protection to the base metal. The fluorinated coatings slowed down the degradation of the Mg alloys during the T0–T1 period. At T1, the mechanical strength of the plates and screws in Group B was similar to that of Group C plates and screws, which was significantly better than that of the control group. However, the mechanical strength of the fluoride-coated plates and screws decreased rapidly as the time of immersion in SBF increased, which can be attributed to the microcracks that appeared on the surface of the fluoride coating under the compressive stress, eventually affecting the protective performance of the fluoride coating (Figure S1). Group C plate and screws coated by duplex MgF_2_/ZrO_2_ showed the most outstanding maintenance performance with respect to mechanical strength. Additionally, Group C plates and screws could maintain more than 30% of its mechanical properties 14 days after immersion in SBF. Combined with the measurement results of micro-CT, the results indicate that ALD ZrO_2_ coating can delay the degradation of the Mg alloy plate–screw system and maintain its early morphological and mechanical stability. Nevertheless, we found that the duplex MgF_2_/ZrO_2_ coating, which maintained the internal fixation structure for the longest time, also fractured within 3 weeks of the onset of degradation, indicating that the protective effect of this coating needs to be further improved.

In vivo experiments are important and need to be supplemented to verify the degradation rate of Mg plates and screws under stress. In addition, the biocompatibility and osteogenesis-promoting properties of different surface coatings also need to be verified by in vivo experiments. Further research is needed to determine whether changing the type of ALD coating, such as HfO_2_ coating, or increasing the thickness of the ALD coating, could further reduce the degradation rate and maintain the mechanical properties of Mg plates and screws under stress conditions.

## 5. Conclusions

A rigid internal fixation system needs to bear some or even all of the physiological stress before the fracture heals. However, studies on the degradation of the Mg alloy plate-screw system under physiological stress conditions are rare. Herein, the degradation and maintenance of the mechanical properties of the rigid internal fixation system of Mg alloys under physiological stress were simulated in vitro.

The degradation rate of different regions of the plate–screw system was inconsistent, and the degradation rate of the screw shaft was significantly slower than that of the screw head and the plate, and there was no significant difference in the degradation rate of the screw shaft under different surface treatment methods (*p* = 0.077). Compared to fluorinated coatings and no surface coating protection, the duplex MgF_2_/ZrO_2_ coating significantly slowed down the degradation rate of the screw head and magnesium plate, which enabled more than 70% volume of the plate and more than 90% volume of the screw head retained after 4 weeks of in vitro experiments. Meanwhile, the mechanical strength of the magnesium alloy plate–screw system protected by the duplex MgF_2_/ZrO_2_ coatings was maintained within 2 weeks during the in vitro degradation process under physiological stress.

According to these findings, it is reasonable to recommend that duplex MgF_2_/ZrO_2_ coated Mg alloy plates and screws can be considered as an alternative choice in the treatment of fractures. However, the length of time it maintains its mechanical properties under stress conditions is less than the time required for bone healing. Hence, the duplex MgF_2_/ZrO_2_ coated Mg alloy rigid internal fixation system should be used with caution for fixing fractures in large stress regions.

## Figures and Tables

**Table 1:** The degradation of plates and screws among the different groups after 4 weeks was assessed by volume quantification.

	Group A	Group B	Group C	*p*
Plate residual (mm^3^)	17.18 ± 2.41	33.01 ± 4.94	43.55 ± 2.12	<0.001 *
Plate residual (%)	30.02	57.68	76.09	
Screw residual (mm^3^)	9.66 ± 0.41	10.65 ± 0.86	12.15 ± 0.55	<0.001 *
Screw residual (%)	73.19	80.69	92.06	
Screw head residual (mm^3^)	1.23 ± 0.30	1.91 ± 0.52	3.17 ± 0.48	<0.001 *
Screw head residual (%)	31.49	48.89	81.14	
Screw shaft residual (mm^3^)	8.43 ± 0.36	8.74 ± 0.89	8.98 ± 0.25	0.077
Screw shaft residual (%)	91.10	94.45	97.04	

* Significant difference between the groups (One-way ANOVA, *p* < 0.05). Group A: without surface coatings; Group B: with the fluorinated coating; Group C: with the duplex fluorinated and ALD ZrO_2_ coatings.

**Table 2:** The mean and standard deviation of loading forces (N) for different vertical displacements among groups.

Group Description	Time Point	1 mm Displacement (N)	3 mm Displacement (N)	5 mm Displacement (N)	10 mm Displacement (N)
No surface coating	T1	0.351 ± 0.079	0.898 ± 0.064	1.632 ± 0.141	3.790 ± 0.397
	T2	0.255 ± 0.051	0.717 ± 0.072	1.428 ± 0.101	2.837 ± 0.267
	T3	0.134 ± 0.061	0.462 ± 0.030	0.931 ± 0.126	1.419 ± 0.161
Fluorinated coating	T1	0.555 ± 0.073	1.593 ± 0.262	2.554 ± 0.247	4.457 ± 0.567
	T2	0.356 ± 0.038	1.059 ± 0.047	1.977 ± 0.252	3.453 ± 0.573
	T3	0.298 ± 0.045	0.687 ± 0.137	1.274 ± 0.116	2.085 ± 0.363
Duplex fluorinated and ALD ZrO_2_ coating	T1	0.556 ± 0.032	1.749 ± 0.265	2.764 ± 0.385	4.687 ± 0.617
	T2	0.514 ± 0.042	1.637 ± 0.221	2.606 ± 0.339	4.223 ± 0.484
	T3	0.489 ± 0.068	1.351 ± 0.283	2.252 ± 0.389	3.474 ± 0.493
	T4	0.276 ± 0.074	0.604 ± 0.133	1.377 ± 0.430	1.983 ± 0.372

## Data Availability

The original contributions presented in the study are included in the article/supplementary material, further inquiries can be directed to the corresponding authors.

## Supplementary Materials

The following supporting information can be downloaded at: https://www.mdpi.com/article/10.3390/ma17143485/s1, Figure S1: Scanning electron microscopy images and energy-dispersive X-ray images of Mg-Zn-Y-Nd alloy without coatings (a,d), fluorinated coatings (b,e), duplex MgF2/ZrO2 coatings (c,f) after 4 weeks immersion test.
