# Supplementary material for: Duplex Fluorinated and Atomic Layer Deposition-Derived ZrO2 Coatings Improve the Corrosion Resistance and Mechanical Properties of Mg-2Zn-0.46Y-0.5Nd (wt.%) Alloy Plates and Screws
Source: Materials (Basel). 2024 Jul 14;17(14):3485. doi: 10.3390/ma17143485 (PMC11278270; doi:10.3390/ma17143485)
Supplement: Supplementary file 1 [file materials-17-03485-s001.zip › materials-3052500-supplementary.pdf]

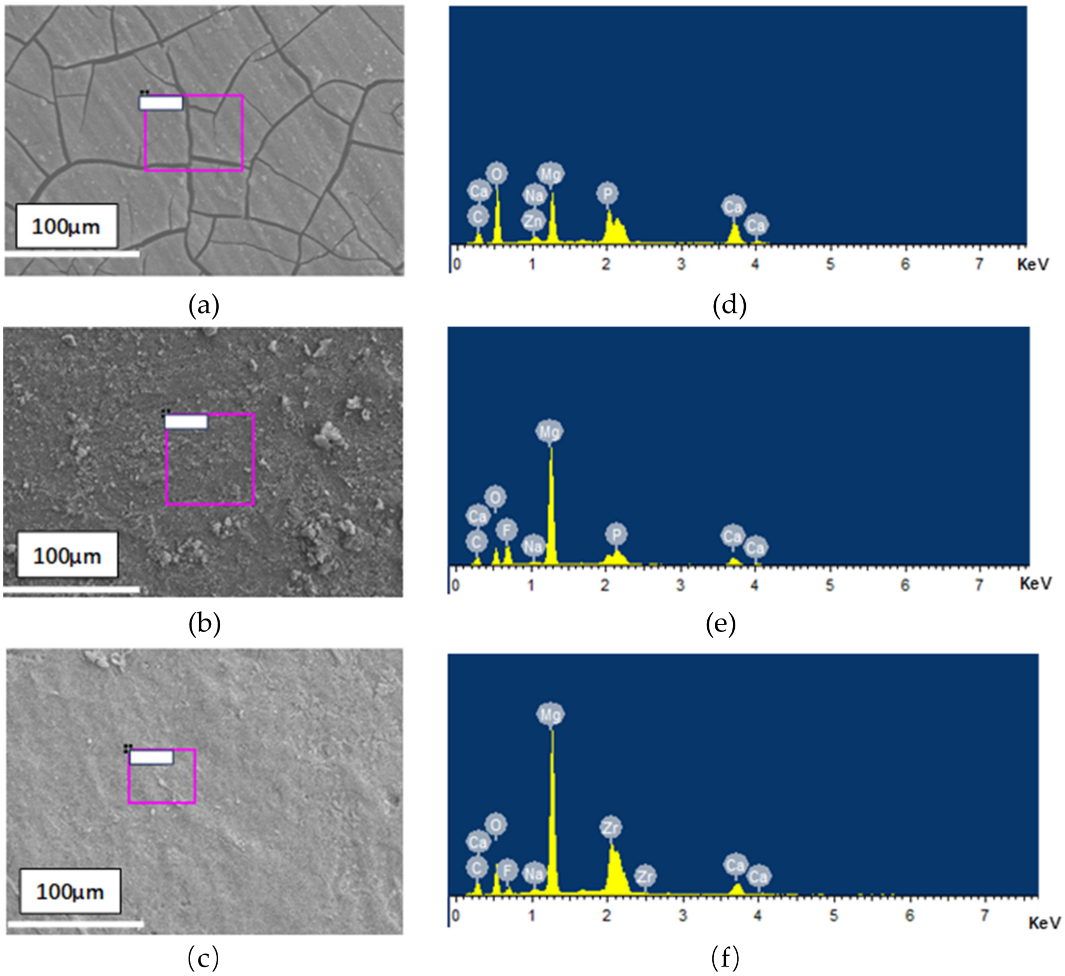

Figure S1. Scanning electron microscopy images and energy-dispersive X-ray images of Mg-Zn-Y-Nd alloy without coatings (a, d), fluorinated coatings (b, e), duplex MgF2/ZrO2 coatings (c, f) after 4 weeks immersion test.
